# Optimization of the CDC Protocol of Molecular Diagnosis of COVID-19 for Timely Diagnosis

**DOI:** 10.3390/diagnostics10050333

**Published:** 2020-05-21

**Authors:** Chao-Ju Chen, Li-Ling Hsieh, Shu-Kai Lin, Chu-Feng Wang, Yi-Hui Huang, Shang-Yi Lin, Po-Liang Lu

**Affiliations:** 1 Department of Laboratory Medicine, Kaohsiung Medical University Hospital, Kaohsiung Medical University, Kaohsiung 80756, Taiwan; chaoju.chen@gmail.com (C.-J.C.); 880163@ms.kmuh.org.tw (L.-L.H.); 900042@ms.kmuh.org.tw (S.-K.L.); 870249@ms.kmuh.org.tw (C.-F.W.); 790207@ms.kmuh.org.tw (Y.-H.H.); 960017@ms.kmuh.org.tw (S.-Y.L.); 2 Department of Internal Medicine, Kaohsiung Medical University Hospital, Kaohsiung 80756, Taiwan; 3 College of Medicine, Kaohsiung Medical University, Kaohsiung 80708, Taiwan

**Keywords:** coronavirus disease 2019 (COVID-19), severe acute respiratory syndrome coronavirus 2 (SARS-CoV-2), molecular diagnostics, real-time reverse transcriptase polymerase chain (RT-PCR)

## Abstract

Coronavirus disease 2019 (COVID-19), the current uncontrolled outbreak of infectious disease, has caused significant challenges throughout the world. A reliable rapid diagnostic test for COVID-19 is demanded worldwide. The real-time reverse transcriptase polymerase chain was one of the most quickly established methods in the novel viral pandemic and was considered as the gold standard for the detection of severe acute respiratory syndrome coronavirus 2 (SARS-CoV-2). In this report, we illustrate our experience of applying a protocol from the Taiwan CDC and achieving assay optimization in the immediate circumstances to meet the urgent medical and public health needs.

In December 2019, a cluster of atypical pneumonia patients of unknown cause emerged in Wuhan, China [1]. A novel beta-coronavirus, currently defined as a severe acute respiratory syndrome coronavirus 2 (SARS-CoV-2) and previously known by the temporary name 2019 novel coronavirus (2019-nCoV), has been identified in these cases [2]. The SARS-CoV-2 caused severe lower respiratory tract infections, referred to coronavirus disease 2019 (COVID-19), which was declared a global public health emergency by the World Health Organization (WHO) on 30 January 2020 and a pandemic on 11 March 2020 [3,4]. There is an urgent need for a rapid and sensitive diagnostic assay to detect COVID-19. In this brief report, we describe how our laboratory, a clinical laboratory in a medical university hospital in Taiwan, set up a COVID-19 molecular diagnosis process rapidly and optimizing the diagnosis assay.

Because testing capabilities are absolutely essential to managing a pandemic, Taiwan Centers for Disease Control (CDC) requested several clinical laboratories to establish COVID-19 testing process and provide urgent laboratory service. As soon as the WHO and U.S. CDC published the protocols for real-time reverse-transcription (RT)-PCR assays [5,6], the Taiwan CDC reorganized a protocol into a Chinese version for each clinical laboratory to adopt rapidly [7]. Due to the urgent and robust demand with laboratory diagnostic testing for COVID-19, our laboratory started to set up real-time RT-PCR assays of COVID-19 on 31 January and has provided clinical service since 3 February 2020.

Respiratory samples (oropharyngeal swabs or sputum) were obtained from patients or contact persons. After sampling, oropharyngeal swabs were placed in viral transport medium, and sputum samples were 1:1 diluted with phosphate buffered saline (PBS) for RNA extraction. Viral RNA was extracted using the RNA purification kit (QIAmp Viral RNA Mini Kit, Qiagen, Germany). According to the protocol suggested by the Taiwan CDC, we performed one-step real-time RT-PCR to detect three different regions (*E* gene, *N* gene and *RdRp* gene) of the viral genome. SARS-CoV RNA (obtained via the European virus archive global [8]) was used as a positive control and RNase-free water was used as negative control for the entire laboratory procedure. RT-PCR reactions were conducted by the LightCycler 480 system (Roche, Mannheim, Germany). The thermal cycling condition was reverse transcribed at 50 °C for 10 min, initial activation at 95 °C for 20 s, 45 cycles of PCR amplification of 5 s at 95 °C, 15 s at 53 °C, and 15 s at 60 °C. The primers and probes used are listed in Table 1. The positive control reaction should be positive at or before 35 cycles, and the negative control should have no fluorescence growth curve which crosses the threshold line. If expected positive or negative control reaction was not achieved, we invalidated the run and repeated the assay. However, during the two weeks after the first PCR test on February 3, we analyzed 477 respiratory samples with no positive results and faced several practical and technical difficulties. We overcame these challenges by optimizing the initial protocol.

Based on our experience, we tried to proportionally reduce half of the volumes of the reagent and compared them with the initial protocol. Three repeats for positive control samples on each target genes were performed, and the Ct values (mean ± SD) were in the similar range in full volume (*E* gene: 26.82 ± 0.02, *R* gene: 25.22 ± 0.02, *N* gene: 24.53 ± 0.03) and half-volume (*E* gene: 26.79 ± 0.06, *R* gene: 25.12 ± 0.04, *N* gene: 24.23 ± 0.01) protocol. Our results did not show a difference in the cycle threshold (*C*_t_) value between the full- and half-volume method (Figure 1). Therefore, we started to use the half-volume method in our routine laboratory service for the COVID-19 test to save our testing materials. The reduction in reagents needed for experiments is important, as a shortage of laboratory supply might happen during the COVID pandemic.

Following the recommendations from the Taiwan CDC, we performed *E* gene and *RdRp* gene as screening and confirmatory assays. If a positive or inconclusive result appeared, *N* gene assay was used as an additional confirmatory assay. At the beginning, we were troubled by the high amounts of nonspecific signals in late cycles of the tested samples and of the negative or blank control sample in the *E* gene assay (Figure 2a). In contrast, the *RdRp* gene assay was free from those nonspecific fluorescence signals in the late cycles of amplification (Figure 2b). We ran DNA electrophoresis for the PCR products from *E* gene assay to verify these nonspecific signals (Figure 2c). In the following, we tried to find a solution to reduce nonspecific signals. As reported in previous studies, bovine serum albumin (BSA) could be an adjuvant that makes the DNA more accessible to the enzyme for amplification, reduces nonspecific binding of primers in CG-rich sequences, and prevents the adhesion of the DNA polymerase to the sides of the capillaries used in certain real-time PCR instruments, such as the LightCycler system [9,10,11]. We observed that incorporation of 0.1 mg/mL of BSA to real-time PCR reactions can greatly help to reduce the nonspecific fluoresce signal (Figure 2d). The event rate of nonspecific signals in *E* gene assay was significantly reduced from 63.1% (301/477) to 12% (226/1890) after adding BSA.

In the earliest stages of the outbreak, the positive rate of SARS-CoV-2 detection by RT-PCR was extremely low. Whereas we performed a positive control and negative control sample in every batch of PCR amplification, it is uncertain whether the process of viral RNA extraction and PCR amplification in each well of the 96-well plate were accomplished successfully. To ensure the whole process of the COVID-19 diagnostic testing took place without a problem, we simultaneously added primers and a probe of human *RNase P* gene (*RP* gene) as an internal control in the same well with the *RdRp* gene assay according to the protocol from the U.S. CDC [6] (Figure 3). The sequences of the primers and probe are listed in Table 1.

The COVID-19 assay from the Taiwan CDC could be rapid and effective if implemented in proficient clinical laboratories, but further optimization, especially of the *E* gene assay and simultaneously with *RP* gene in each reaction, may enhance the confidence in laboratory diagnosis. The optimized protocol and workflow are displayed in Figure 4. By 31 March, we analyzed 1890 respiratory samples using optimized protocol and 8 of them showed positive results for SARS-CoV-2 RNA. Whereas some useful molecular-based point-of-care testing systems for COVID-19 testing, such as ID NOW™ (Abbott, San Diego, CA, USA) and GeneXpert^®^ (Cepheid, Sunnyvale, CA, USA), were developed and received Food and Drug Administration (FDA) emergency-use authorization (EUA) [12], the current standard approach for COVID-19 diagnosis still requires a real-time RT-PCR assay. Moreover, in the early stages of the epidemic, the laboratory developed an RT-PCR assay that was the most practical and reliable laboratory method in diagnostic virology based on currently available technology. In this pandemic, timely and effective RT-PCR assay design was enabled by the availability of significant sequence knowledge from many years of investigation of SARS-related viruses in animal reservoirs since 2003 [13]. In the present report, we demonstrated our experience of relying on a protocol template from the Taiwan CDC to establish an optimized COVID-19 molecular diagnostic test within our routine services in a public health emergency.

## Figures and Tables

**Figure 1:**
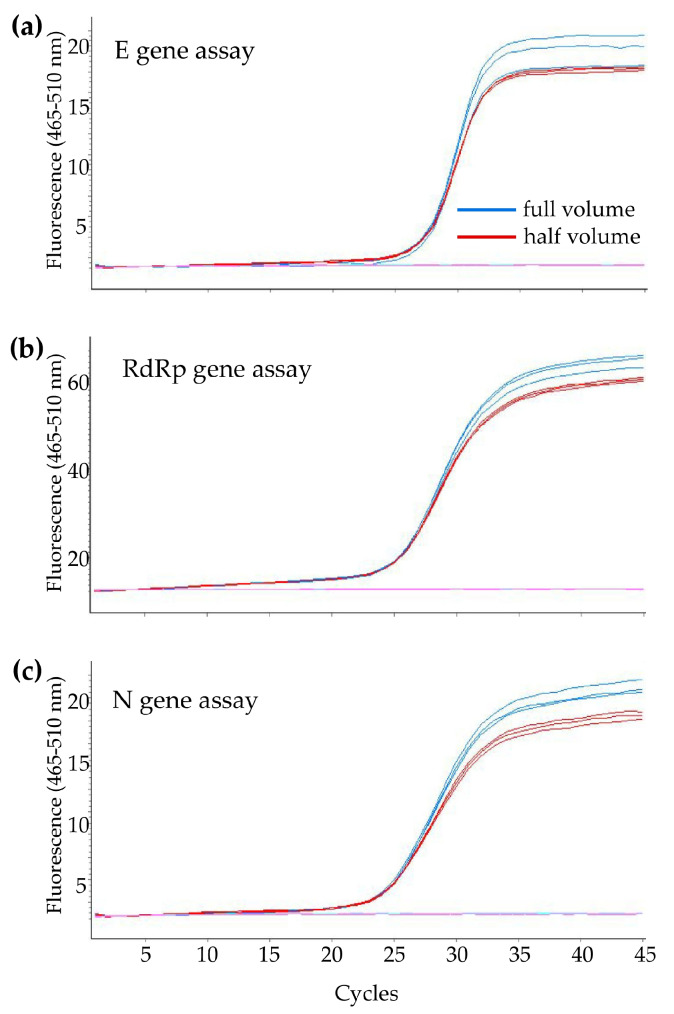
Example for amplification curves of the (**a**) *E* gene assay, (**b**) *RdRp* gene assay, and (**c**) *N* gene assay with full-volume (blue curves) and half-volume (red curves) reagents.

**Figure 2:**
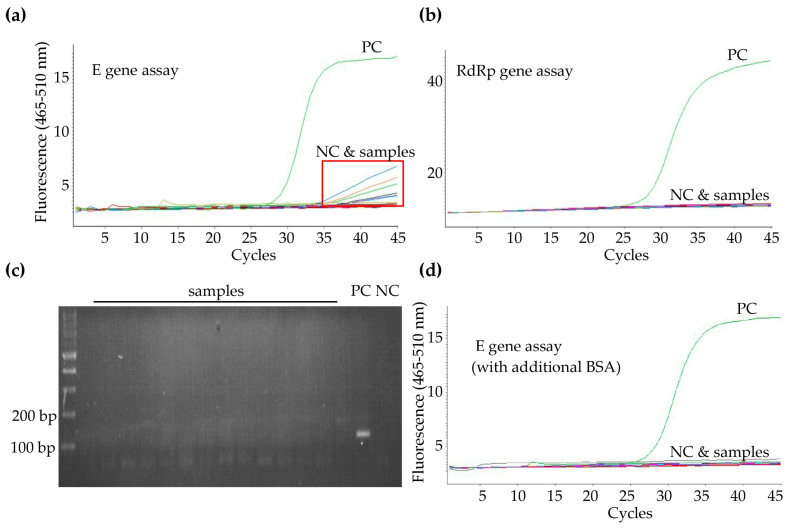
Verified and solved nonspecific fluorescent signal for *E* gene assay. (**a**) Nonspecific fluorescence signal in the late cycles of late cycles (red rectangle) in *E* gene assay; (**b**) Amplification curve without nonspecific fluorescence signal in *RdRp* gene assay; (**c**) Verified the PCR products of *E* gene assay by gel electrophoresis and only the positive control sample showed a band with the correct size (expected 112 bp). (**d**) The nonspecific fluorescence signal reduced after adding 1 mg/mL of bovine serum albumin (BSA) to the reverse transcriptase polymerase chain reactions (PCR).

**Figure 3:**
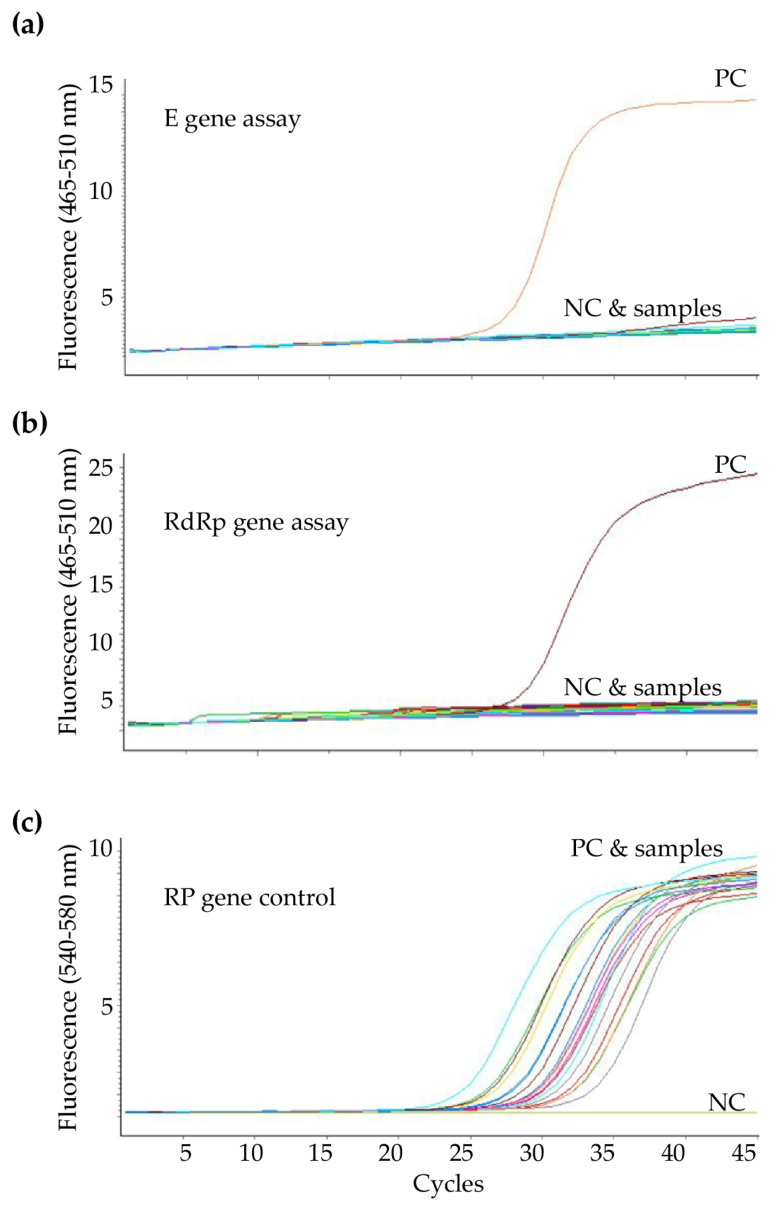
Example for amplification curves of the (**a**) *E* gene assay and (**b**) *RdRp* gene assay with (**c**) *RP* gene assay using the optimized real-time RT-PCR protocol.

**Figure 4:**
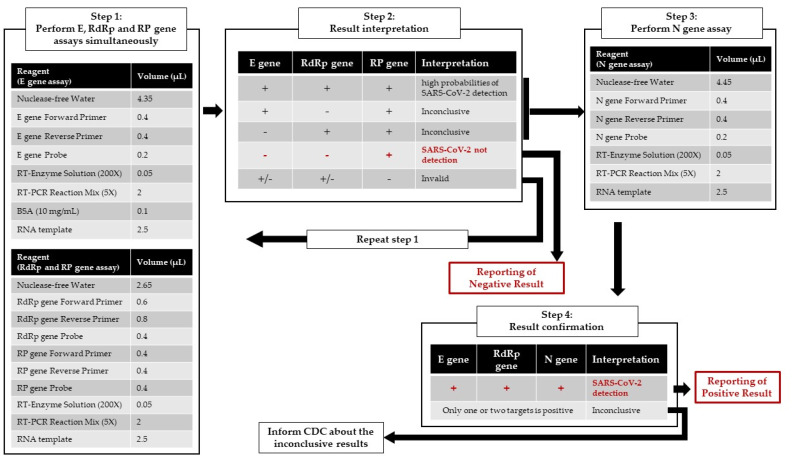
Flowchart of the optimized COVID-19 molecular diagnostic workflow protocol in the laboratory at the Kaohsiung Medical University Hospital.

**Table 1:** 2019-Novel Coronavirus (SARS-CoV-2) Real-Time RT-PCR Panel Primers and Probes.

Description	Oligonucleotide Sequence (5′ > 3′)	Reference
*E* gene F’	ACAGGTACGTTAATAGTTAATAGCGT	[5]
*E* gene R’	ATATTGCAGCAGTACGCACACA	
*E* gene probe	FAM-ACACTAGCCATCCTTACTGCGCTTCG-BBQ	
*RdRp* gene F’	GTGARATGGTCATGTGTGGCGG	[5]
*RdRp* gene R’	CARATGTTAAASACACTATTAGCATA	
*RdRp* gene probe	FAM-CAGGTGGAACCTCATCAGGAGATGC-BBQ	
*N* gene F’	CACATTGGCACCCGCAATC	[5]
*N* gene R’	GAGGAACGAGAAGAGGCTTG	
*N* gene probe	FAM-ACTTCCTCAAGGAACAACATTGCCA-BBQ	
*RP* gene F’	AGATTTGGACCTGCGAGCG	[6]
*RP* gene R’	GAGCGGCTGTCTCCACAAGT	
*RP* gene probe	HEX-TTCTGACCTGAAGGCTCTGCGCG-BHQ	

## References

[1] Zhu N., Zhang D., Wang W., Li X., Yang B., Song J., Zhao X., Huang B., Shi W., Lu R. (2020). A Novel Coronavirus from Patients with Pneumonia in China, 2019. N. Engl. J. Med..

[2] Gorbalenya A.E., Baker S.C., Baric R.S., de Groot R.J., Drosten C., Gulyaeva A.A., Haagmans B.L., Lauber C., Leontovich A.M., Neuman B.W. (2020). The species Severe acute respiratory syndrome-related coronavirus: Classifying 2019-nCoV and naming it SARS-CoV-2. Nat. Microbiol..

[3] WHO (2020). Coronavirus Disease 2019 (COVID-19) Situation Report—53.

[4] WHO (2020). Coronavirus Disease 2019 (COVID-19) Situation Report—10.

[5] Diagnostic Detection of Wuhan Coronavirus 2019 by Real-Time RT-PCR. https://www.who.int/docs/default-source/coronaviruse/wuhan-virus-assay-v1991527e5122341d99287a1b17c111902.pdf.

[6] 2019-Novel Coronavirus (2019-nCoV) Real-Time rRT-PCR Panel Primers and Probes. https://www.cdc.gov/coronavirus/2019-ncov/lab/rt-pcr-panel-primer-probes.html.

[7] Taiwan CDC 2019-nCoV Virus Nucleic Acid Test. https://www.cdc.gov.tw/File/Get/BIHQoIEBjlFZ5tsjfij2Gg.

[8] Mahase E. (2020). China coronavirus: WHO declares international emergency as death toll exceeds 200. BMJ.

[9] Nagai M., Yoshida A., Sato N. (1998). Additive effects of bovine serum albumin, dithiothreitol, and glycerol on PCR. Biochem. Mol. Biol. Int..

[10] Nitsche A., Steuer N., Schmidt C.A., Landt O., Siegert W. (1999). Different real-time PCR formats compared for the quantitative detection of human cytomegalovirus DNA. Clin. Chem..

[11] Plante D., Bélanger G., Leblanc D., Ward P., Houde A., Trottier Y.L. (2011). The use of bovine serum albumin to improve the RT-qPCR detection of foodborne viruses rinsed from vegetable surfaces. Lett. Appl. Microbiol..

[12] Yang T., Wang Y.C., Shen C.F., Cheng C.M. (2020). Point-of-Care RNA-Based Diagnostic Device for COVID-19. Diagnostics (Basel).

[13] Decaro N., Lorusso A. (2020). Novel human coronavirus (SARS-CoV-2): A lesson from animal coronaviruses. Vet. Microbiol..

